# Clinical and Organizational Issues in the Management of Surviving Breast and Colorectal Cancer Patients: Attitudes and Feelings of Medical Oncologists

**DOI:** 10.1371/journal.pone.0101170

**Published:** 2014-07-01

**Authors:** Gianmauro Numico, Carmine Pinto, Stefania Gori, Giovanni Ucci, Massimo Di Maio, Maurizio Cancian, Francesco De Lorenzo, Nicola Silvestris

**Affiliations:** 1 Medical Oncology Unit, Azienda USL della Valle d′Aosta, Aosta, Italy; 2 Medical Oncology Unit, S. Orsola-Malpighi Hospital, Bologna, Italy; 3 Medical Oncology, Sacro Cuore Don Calabria Hospital, Verona, Italy; 4 Oncology Department, Azienda Ospedaliera di Lodi, Lodi, Italy; 5 Clinical Trial Unit, National Cancer Institute “G. Pascale” Foundation, Napoli, Italy; 6 Società Italiana di Medicina Generale (SIMG), Conegliano, Italy; 7 Federazione Associazioni di Volontariato in Oncologia (FAVO), Roma, Italy; 8 Medical Oncology Unit, National Cancer Research Centre “Giovanni Paolo II”, Bari, Italy; University of Bari & Consorzio Mario Negri Sud, Italy

## Abstract

**Background:**

The fast growing demand and the shortage of resources are pushing toward more efficient models of survivorship care delivery. The Associazione Italiana di Oncologia Medica (AIOM) established an interdisciplinary working group with the purpose of promoting organizational improvements at the national level. A survey aimed at assessing attitudes and feelings of oncologists was considered preliminary to further initiatives.

**Methods:**

A 25-item questionnaire, sent to the mailing list of the Society, explored the following issues on the practice of breast and colorectal cancer patients' follow up: 1) organization; 2) clinical features; 3) feelings about the different meanings of follow-up.

**Results:**

Ninety-one oncologists of 160 institutions (57%) answered to the questionnaire. Although follow up is considered a distinct oncological activity in 68%, a fully shared organization between specialists is not common and communications with Primary Care Physicians are not structured in the majority of the cases. Fifty-five and 30% of the oncologists follow breast and colorectal cancer patients indefinitely. In case of discharge a survivorship care plan is delivered in only 9%. The majority of respondents do not hold a role of follow up in mortality reduction.

**Conclusions:**

Although survivorship care represents a significant part of the oncologists' workload, an “oncology-centered” model is largely adopted and established care pathways are still incomplete. Survivorship care needs to be put at the center of an educational policy and of a widespread organizational effort, directed at improving appropriateness and quality.

## Background

The number of people surviving to cancer is rapidly increasing and accounts approximately for the 6% of the adult population in developed countries [1], [2]. Although some patient with a cancer history comes back to a completely healthy state [3] the majority is subject to disease and treatment-related conditions, is at risk of relapse and of other cancer and non-cancer diseases. The medical needs of cancer survivors are long-lasting: in most primaries clinical research has not been able to find clear time cutoffs after which the risk of relapse becomes comparable to that of the general population [4]–[6]. Moreover, some clinical conditions, such as anthracycline-related cardiotoxicity or secondary tumors are typically detected many years after the primary cancer [7], [8]. Survivors are thus carriers of multiple medical needs and survivorship care is becoming a requested, multidimensional medical activity [9]. However, the increasing prevalence of cancer and the shortage of economical and human resources [10]–[13] is making critical the delivery of appropriate care.

An evolution of organizational models of care delivery, the sharing of medical skills and the promotion of definite and cost-effective care pathways are thus considered crucial issues [14]. Randomized trials have shown that efficient models of survivorship care can be introduced in clinical practice without impairing the quality of assistance and patients satisfaction: PCP-based follow up [15]–[17] nurse-based follow up [18] or simplified specialist schedules [19], [20].

In the Italian context, from the usually adopted “oncology centered” model, a “sequential” model is gaining acceptance: after an arbitrarily defined period of specialist follow up, patients are discharged and sent to the PCP. While the sequential model is an easy way of discharging patients, it would imply a detailed transmission of clinical informations and the availability of efficient ways of re-enter in the specialist circuit in case of suspected relapse or complex conditions. A proposed alternative is the “shared cares” model [14] that promotes a continuous interaction between specialists and PCP throughout the whole clinical history with different roles of clinical actors in different times. This model requires high quality interactions, definite procedures and above all a well-defined communication infrastructure consisting in shared medical records.

Whatever the model, a new attitude to share competences and practices is required to the medical oncologist, together with an organizational effort aimed at maintaining quality and improving the efficiency of survivorship care. The main issues to be evaluated are: 1) the peculiar requirements of follow-up consultations in terms of know-how and organization. 2) the interaction with other specialists involved in survivorship care (surgeons and radiotherapists among all). 3) the appropriateness of medical procedures. 4) the relationship with PCP. All these aspects are influenced by deep and personal beliefs about the purposes of follow-up consultations.

We were interested in going into both the feelings and practical solutions adopted by Italian medical oncologists: an improvement in this field could hardly be a matter of the single center or the single oncologist. Rather, attitudes, ideas and obstacles are widespread at a national level as strictly reflect the overall quality of the national health care system. Having a real picture of how oncologists deal with survivorship care was considered a necessary preliminary step for further suggestion of possible organizational improvements.

## Methods

The “Associazione Italiana di Oncologia Medica” (AIOM), in recent years established a working group with the aim of studying the issues related to the practice of survivorship care and of making proposals about possible organizational improvements. The working group is composed of medical and radiation oncologists, PCPs and representatives of patients advocacy associations. Due to the heterogeneity of follow up practices among oncologists and the lack of standardized procedures, it was decided that the first objective of the group should have been a thorough knowledge of feelings and attitudes of medical oncologists about survivorship care. A 25-item, questionnaire was sent three times to the mailing list of the Society, in a 2-months period. One oncologist per institution was asked to anonymously answer to the questions. After an introductory section dealing with institution characteristics, the following sections were presented: 1) organizational aspects of follow-up consultations; 2) clinical aspects involving breast and colorectal cancer follow-up; 3) feelings of medical oncologists about various meanings of follow-up.

According to the National regulations no Ethical Committee approval was deemed necessary, given the absence of data about patients. Instead, a privacy statement was provided to every responding oncologist.

The data entry was done by AIOM and descriptive statistics of the data were performed used Excel software (Office 2010). After the analysis, the results were discussed in the working group and further activity was planned accordingly.

## Results

Ninety-one medical oncologists of the 160 Italian institutions answered to the questionnaire (response rate 57%). Characteristics of the answering oncologists and their institutions are shown in table 1. There was a prevalence of northern centers (58%) and of general hospitals (75%). Among respondents 41% were heads of department. The majority of the respondents take care of more than 100 and less than 100 new breast and colorectal cancer patients every year respectively.

**Table 1:** Characteristics of responding oncologists and institutions.

	N	%
**Geographic area**		
Northern	53	58%
Central	14	15%
Southern and major isles (Sardinia, Sicily)	20	22%
Not reported	4	4%
**Position of the oncologist**		
Head of department	37	41%
Other role	54	59%
**Institution**		
General Hospital	68	75%
University Hospital	14	15%
Mixed	3	3%
Private	6	7%
**Number of beds of the institution**		
0–300	37	41%
300–600	34	37%
>600	13	14%
Not reported	7	8%
**Number of oncologists in the department**		
1–5	26	29%
6–10	42	46%
>10	21	23%
Not reported	2	2%
**Organization of the oncology department**		
Only Day Hospital/ambulatory service available	33	36%
Beds available	58	64%
**Number of new cancer patients seen yearly**		
100–400	19	21%
400–1000	39	43%
>1000	14	15%
Not reported	19	21%
**Number of new breast cancer patients seen yearly**		
20–100	32	35%
100–200	25	27%
>200	29	32%
Not reported	5	6%
**Number of new colorectal cancer patients seen yearly**		
20–100	49	54%
100–200	21	23%
>200	14	15%
Not reported	7	8%

### Organization of follow-up

The complete list of questions and answers is shown in table 2. For 62 (68%) oncologists follow up represents a distinct medical activity, with dedicated times and waiting list, while for 29 (32%) follow up consultations are mixed with those of patients with active disease. The duration of the follow up consultation is 20 minutes or less in 64%. The estimated weekly time dedicated to follow up is between 8 and 32 hours in 69% of cases.

**Table 2:** Questionnaire items: organizational features.

Question	Answers	N	%
Do you have dedicated follow-up ambulatories?	Yes	62	68%
	No	29	32%
How much time do you spend for the follow-up visit?	Less than 20 min	14	15%
	20 min	45	49%
	30 min	19	21%
	Variable	9	10%
	Not stated	4	4%
How many hours a week are spent for follow-up visits in your institution?	Less than 8	15	16%
	8–16	34	37%
	16–32	29	32%
	More than 32	13	14%
Is there an agreement between specialists for the rationalization of follow-up? Breast Cancer (1 not applicable)	Yes	32	36%
	No formal agreement but some form of alternation of the visits	32	36%
	No	26	29%
Is there an agreement between specialists for the rationalization of follow-up? Colorectal Cancer (1 not applicable)	Yes	36	40%
	No formal agreement but some form of alternation of the visits	12	13%
	No	42	47%
Is the medical record shared by all the specialists? Breast cancer (1 not applicable)	Yes	17	19%
	No	55	61%
	No but informations are available	18	20%
Is the medical record shared by all the specialists? Colorectal cancer (1 not applicable)	Yes	10	11%
	No	59	66%
	No but informations are available	21	23%

Some form of alternation of the visits of the various specialists that are involved in follow up is applied in 36% in breast cancer follow up and 13% in colorectal cancer follow up, while a fully shared organization is reported in 36% and 40% respectively. However, a shared medical record is used in only 19% and 11% of the respondents respectively.

Medical oncologists refer patients to their PCP for booking next consultations in 56% of the cases, while an autonomous request is filled in 34% (Table 3). The figures referring to laboratory and imaging are 54% and 38% respectively. In case of suspicious signs or symptoms occurring between programmed appointments, an accelerated referral to the oncological center is possible in 85%. The ordinary communication between the oncologist and the PCPs consists in the medical report released to the patient (87%) and only rarely in other instruments (dedicated phone line, booklets or dedicated informative material, 8%). Also, when the patient is discharged from the oncological follow up, a conclusive letter is sent to the PCP in 74% of the cases, while a full survivorship care plan or other informative material is delivered only in 9%. When is asked to the oncologists what should be the role of PCP in their opinion the answer is that it should be better exploited in the majority of the cases (89%) and only 4% answer that it is useless or irrelevant.

**Table 3:** Questionnaire items: relationships with the Primary Care Physician (PCP).

Question	Answers	N	%
Who fills the request form of the follow up visit?	Oncologist	31	34%
	PCP	51	56%
	Both	9	10%
The reservation of the next visit*:	Is provided by the oncologist	52	57%
	Is made by the patient at the Hospital booking center	47	52%
	is possible by phone or internet	35	38%
Laboratory and imaging examination are requested and booked by:	Oncologist	35	38%
	PCP	49	54%
	Both	7	8%
If the patient has a warning sign or symptom between the follow up visits*:	Can refer to the Oncologist through an urgent reservation	77	85%
	Refers to the PCP for the first level diagnostic work-up	12	13%
	Refers to the emergency department	9	10%
	Waits for the next visit	0	0
What instruments are used to communicate with the PCP during follow up?	Report of the consultation given to the patient (only)	79	87%
	Additional informative booklets dealing with survivorship care	4	4%
	Additional dedicated telephone line	4	4%
	No communication is planned	4	4%
Is there a survivorship care plan to be delivered to the PCP at the end of the oncological follow up?	Only report of the discharge visit given to the patient	67	74%
	Informative booklets dealing with survivorship care	8	9%
	no communication is planned	16	17%
What do you think about the role of the PCP in follow up?	Is essential	6	7%
	Should be better exploited	81	89%
	Is irrelevant	1	1%
	It adds a useless step in survivorship care	3	3%

*More than one choice was possible, thus the sum being more than 91.

### Clinical aspects of follow up (Table 4)

The majority of oncologists report to adopt institutional guidelines for follow up (74% breast, 77% colorectal cancer). 55% say to follow breast cancer patients indefinitely while 45% establish a time after which the patient is discharged to the PCP: for 38% the cutoff is 5 years and for 7% is 10 years after primary treatment. Colorectal cancer patients are followed indefinitely by 30% of the oncologists while in 70% a cutoff is established (at 2–3 years in 2%, at 5 years in 62% and at 10 years in 4%).

**Table 4:** Questionnaire items: clinical issues.

Question	Answers	N	%
Do you use internal/institutional guidelines? Breast cancer (1 not applicable)	Yes	67	74%
	No	23	26%
Do you use internal/institutional guidelines? Colorectal cancer (1 not applicable)	Yes	69	77%
	No	21	23%
For how much time you follow your patients? Breast cancer (2 not applicable)	2–3 years	0	0
	5 years	34	38%
	10 years	6	7%
	Indefinitely	50	55%
For how much time you follow your patients? Colorectal cancer (2 not applicable)	2–3 years	2	2%
	5 years	55	62%
	10 years	4	4%
	Indefinitely	27	30%
Do you usually perform a thorough physical examination? Breast cancer (1 not applicable)	Yes	81	90%
	No	1	1%
	Only if clinically indicated	8	9%
Do you usually perform a thorough physical examination? Colorectal cancer (1 not applicable)	Yes	76	84%
	No	3	3%
	Only if clinically indicated	11	23%

**Figure 1:**
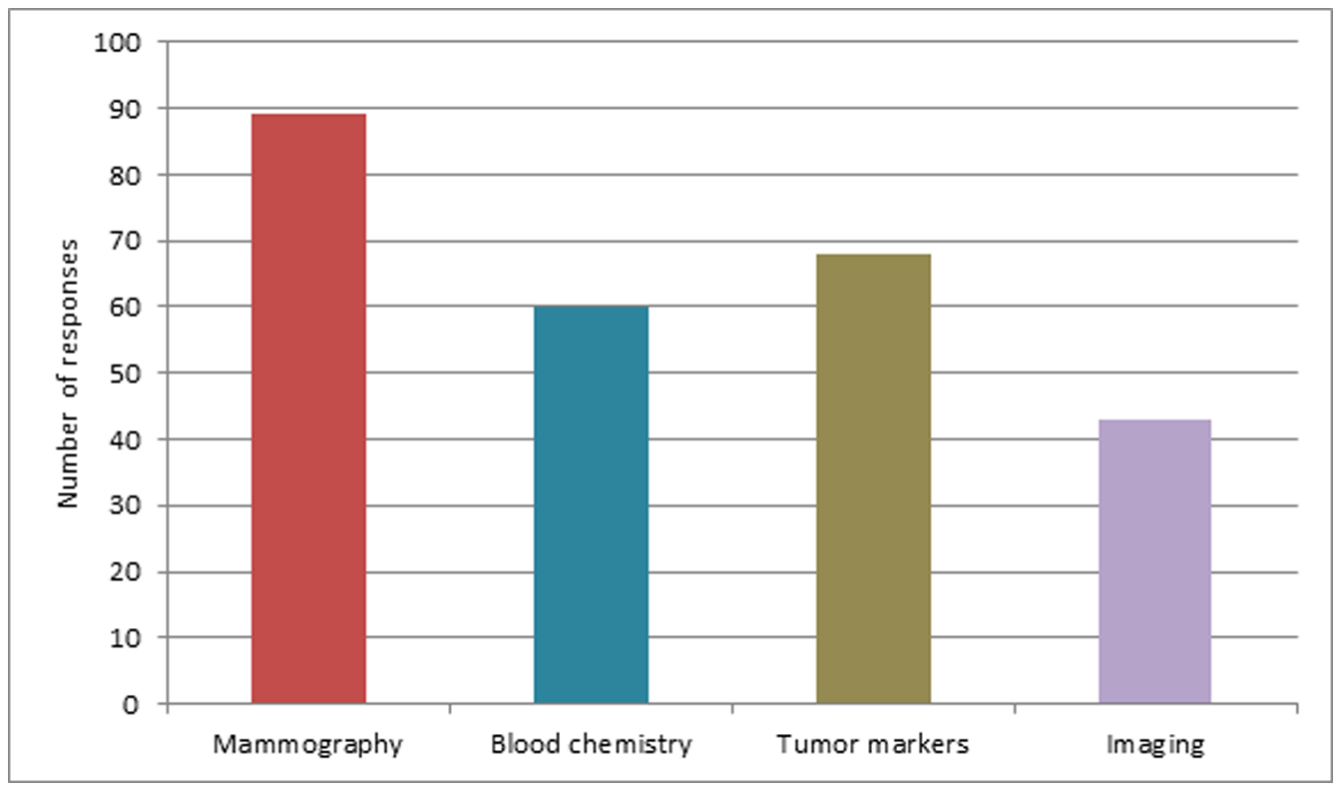
Which exams do you ask independently from clinical findings with the purpose of early diagnosis of relapse? Breast Cancer.

**Figure 2:**
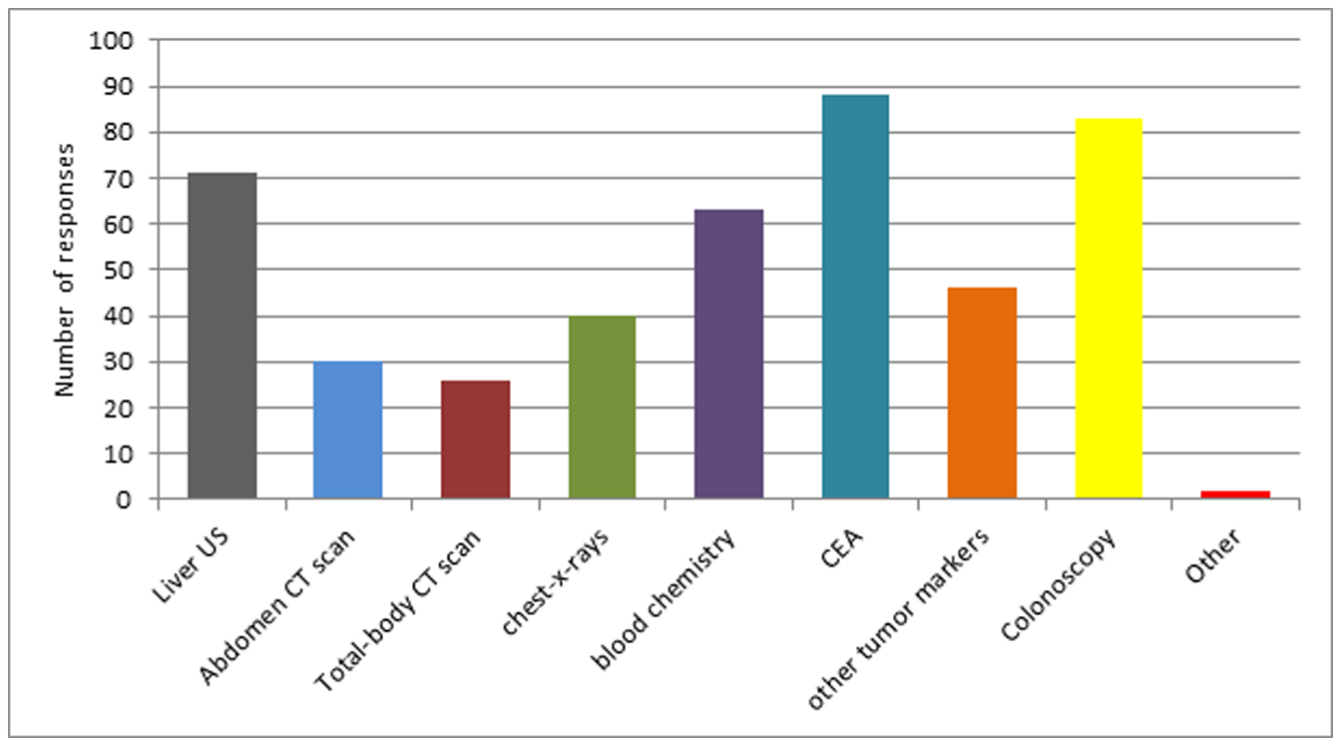
Which exams do you ask independently from clinical findings with the purpose of early diagnosis of relapse? Colorectal cancer.

The general physical exam is considered useful and performed by 81% and 76% of the oncologists for breast and colorectal cancer patients respectively. Among the examinations requested independently from clinical findings, 47% of the oncologists claim to order imaging, 76% order tumor markers and 66% order biochemistry not recommended by any breast cancer follow up guideline (Figure 1). For colorectal cancer follow up, requested inappropriate tests are tumor markers other than CEA (requested by 50%) and biochemistry (requested by 69%) (Figure 2).

### Perceived meaning of follow up

**Figure 3:**
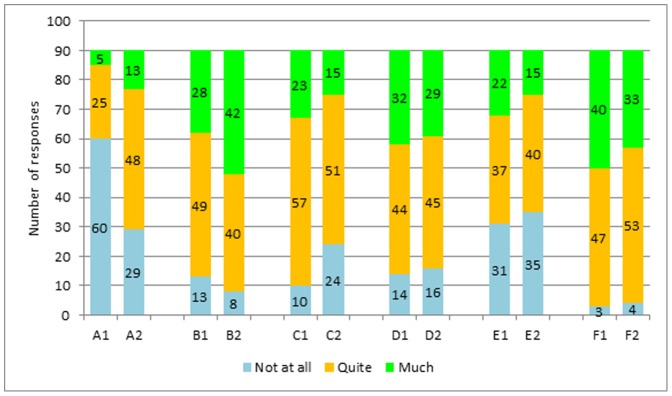
Do you agree that follow up has an important role in A: mortality reduction; B: reduction of the risk of relapse; C: management of late toxicities; D: education and life style change; E: management of comorbidities; F: psychological support. 1: breast cancer; 2: colorectal cancer.

The final part of the survey assessed the meaning of follow up as perceived by oncologists (Figure 3). Only 6% and 14% of the oncologists feel that follow up has an important role in reducing mortality or anticipating the detection of recurrences in breast cancer. These figures grow to 31% and 47% for colorectal cancer. An important role for management of late toxicities is perceived by 26% for breast cancer and 17% for colorectal cancer. Management of comorbidities is perceived a major role in survivorship care by 24% for breast and 17% for colorectal cancer. The educational role is considered important in 36% and 32% while the psychological and supportive role for 44% and 37%.

## Discussion

Follow up is an increasing part of oncologists working time. Although the estimated weekly time dedicated to this practice is reported to be between 8 and 32 hours in almost 70% of the cases, in 32% the follow up consultation has not a specific organization and a dedicated time, separated from consultations of patients with active disease, leaving the recognition of related workload largely underestimated.

It is generally recognized that follow up consultations have distinct characteristics, that differ from other types of medical consultations: 1) they are a “low-efficiency” activity: many patients are to be seen in order to detect a single event; 2) they are quite simple, being clinical examination and few, usually well-defined laboratory or imaging studies routinely requested; 3) the supportive and educational role of the physician are crucial. Actually physical examination is ordinarily performed and the time employed for a single visit is usually brief (20 minutes or less in 64%). All these features would suggest that survivorship care be applied in a specific organizational context: while it seems to be the case for the majority of Italian oncologists, AIOM supports more effort in improving efficiency of this practice, being the shortage of oncological workforce an impending issue [11]–[13].

Another issue highlighted by the survey is the quality of oncological surveillance and the appropriateness of diagnostic tests in asymptomatic patients. As already reported in other contexts, “cancer patients commonly undergo much more intensive surveillance than is commonly recommended by guidelines” [21]–[23]. Although discouraged by available guidelines, more than half of the surveyed oncologists ask for tumor markers in breast cancer, markers other than CEA and blood chemistry in colorectal cancer (figure 1). This finding may be the result of insufficient knowledge and unreliable data on cancer surveillance in breast and colorectal cancer: recommendations are based on old studies and do not consider the different risk of disease subgroups [24]–[25]. However, given the unproven benefits of a more intensive follow up, a strict and uniform adherence to guidelines should be pursued by our Society.

As expected, the model of care delivery is not homogeneous among Italian oncologists. More than half of them claim to take care indefinitely of breast cancer patients; the percentage is lower for colorectal cancer (30%) but stands for a still rooted “oncology centered” model. Although the “sequential” model (specialist phase followed by PCP phase) is frequently adopted, the survey highlighted organizational flaws in the transition process. Before discharge, the main problems seem to be a scarce interaction among specialists (that often independently schedule consultations and examinations and do not use a common medical record) and undefined modalities of booking appointments (with the PCP often involved as an administrative prescriber of clinical or instrumental examinations decided elsewhere). All these issues possibly result in redundancy and inefficiency and are cause of patients' discomfort and confusion. The transition from specialist to PCP care does not seem to be fully managed: a survivorship care plan is released in less than 10% of the cases and only the report of the last consultation is sent to the PCP in the majority of the cases. Although a recent randomized trial did not demonstrate a clear impact on the quality of care [26], [27], survivorship care plans have been strongly recommended by international institutions [28], [29], [21]–[23] and are considered a key tool for a simple management of the informations' flow. The transition of care should be planned in detail and bidirectional communications should be clear and informative. The 2005 Institute of Medicine report recommended the use of survivorship care plans as a tool to help patients and PCP providing appropriate care and prevent from discontinuing care when specialist follow up ends [28].

Actually the large majority of oncologists consider important the role of PCP or state that his/her function should be better exploited. This has not been the case in the recently reported American survey on breast cancer, in which more than two-thirds of oncologists do not think that PCP have the necessary skills to provide follow up care and 58% of the oncologists consequently favor an “oncology centered” model [30].

The perceived meaning of follow up as a survival-improving practice is another important issue: two-thirds and one-third of the surveyed oncologists do not consider relevant the impact on survival of follow up practices in breast and colorectal cancer respectively (figure 2). This finding is in line with the available informations: accumulating evidences suggest a limited role of breast cancer follow up [31], while patients with a history of colorectal cancer probably have some survival benefit [32]. For all of the other items the role of surveillance is perceived more important, with the highest values achieved for the psychological and supportive role. A growing propensity of thinking at follow up as a practice that involves global care of the surviving patient is probably a message of the survey.

Inherent limitations of our data must be taken into account in interpreting the results. A selection bias is likely: the respondents are probably those already involved in the reflection and organizational effort about survivorship care and thus may represent the more sensible component of Italian medical oncology in this field. This could have resulted in an overestimation of the quality of oncological surveillance. Also, the limited number of respondents, although probably representative of the oncological community, may not capture the real-world practice. Finally, what physicians declare may not reflect what is really done in the everyday activity. However, the survey was not intended to be an exact image of survivorship care in Italy, but rather an inquiry about the feelings and sensibility of oncologists. From this point of view the reported data support an active role of AIOM in inspiring a cultural debate and in designing best practice models.

## Conclusions

Follow up is an important and increasing practice for medical oncologists. While there are areas of huge inappropriateness that should be dealt with, the transition to a real survivorship care should be encouraged and promoted in order to cover the needs of global care of the patients with cancer history.
